# Supplementary material for: Imperceptible energy harvesting device and biomedical sensor based on ultraflexible ferroelectric transducers and organic diodes
Source: Nat Commun. 2021 Apr 23;12:2399. doi: 10.1038/s41467-021-22663-6 (PMC8065095; doi:10.1038/s41467-021-22663-6)
Supplement: Supplementary file 1 — Supplementary Information [file 41467_2021_22663_MOESM1_ESM.pdf]

Supplementary Information (SI)

Imperceptible energy harvesting device and biomedical sensor based on ultraflexible ferroelectric transducers and organic diodes

Andreas Petritz[1,2], Esther Karner-Petritz[1,2], Takafumi Uemura[1,3], Philipp Schäffner[2], Teppei Araki[1,3], Barbara Stadlober[2,], Tsuyoshi Sekitani[1,3,]**

[1]Institute of Scientific and Industrial Research
Osaka University, Ibaraki
Osaka 567-0047, Japan
E-mail: sekitani@sanken.osaka-u-ac.jp

[2]JOANNEUM RESEARCH Forschungsgesellschaft mbH
MATERIALS-Institute for Surface Technologies and Photonics
Franz-Pichler Straße 30
8160 Weiz, Austria
E-mail: barbara.stadlober@joanneum.at

[3]AIST Advanced Photo-Bio Lab
Photonics Center Osaka University P3 Bldg.2-1
Yamada-Oka, Suita
Osaka 565-0871, Japan
E-mail: sekitani@sanken.osaka-u-ac.jp

Table of contents

Supplementary Note 1	Ferroelectric-to-paraelectric phase transition	3
Supplementary Note 2	Organic diode: Vertical Schottky setup	3
Supplementary Note 3	Estimation of energy harnessing performance from biomechanical motion of multi-layer UFPTs	5
Supplementary Note 4	Calculation of the UFPT's energy conversion efficiency	6
Supplementary Figure 1	Schematic procedure for 'poling' of a ferroelectric transducer	11
Supplementary Figure 2	Ferroelectric-to-paraelectric phase transition	12
Supplementary Figure 3a	AFM-images of P(VDF:TrFE) films for different annealing temperatures	13
Supplementary Figure 3b	AFM-images of P(VDF:TrFE) films for different annealing temperatures	14
Supplementary Figure 4	XRD-pattern of P(VDF:TrFE) films for different annealing temperatures	15
Supplementary Figure 5	Determination of crystallinity X_c	16
Supplementary Figure 6	Transversal load tests on various substrates	17
Supplementary Figure 7	FEM simulation of the transversal load test on elastic rubber carrier	18
Supplementary Figure 8	Transversal load tests: Multiple layers	19
Supplementary Figure 9	Transversal load tests: Multiple layers	19
Supplementary Figure 10	Strain tests	20
Supplementary Figure 11	Pulse wave measurements on the wrist	21
Supplementary Figure 12	Organic diode: Vertical Schottky setup	22
Supplementary Figure 13	OTFT-based organic diodes	23
Supplementary Figure 14	Ultraflexible energy harvesting device (UEHD)	24
Supplementary Figure 15	Energy harvesting: Energy output by periodic manual bending of one UFPT (mode A)	25
Supplementary Figure 16	Energy harvesting: UFPT bending tests on rail (mode B)	26
Supplementary Figure 17	Energy harvesting: Continuous pressing with a fingertip on the bent transducer (mode C)	27
Supplementary Figure 18	Thin-film capacitor	27
Supplementary Figure 19	FEM simulation of the bending actuation (mode B)	28
Supplementary Figure 20	Energy conversion efficiency of the UFPT (Mode B)	28
Supplementary Table 1	Transducer charge response under transversal load for the three excitation scenarios	29
Supplementary Table 2	Geometry and material parameters used for FEM analysis	29
Supplementary Table 3	Comparison of the performance parameter of vertical diodes and OTFT-based diodes, both with DNTT as the active semiconducting layer	30
Supplementary References		31

Supplementary Notes

Supplementary Note 1: Ferroelectric-to-paraelectric phase transition

As expected, we observe a clear ferroelectric-to-paraelectric phase transition in our ultraflexible ferroelectric polymer transducer, which appears in Supplementary Fig. 2a as a peak in the temperature behaviour of the relative permittivity ε_r *(T)*[1]. At a temperature of 105 °C, the permittivity exhibits a maximum (ε_r ~ 40) that corresponds to the complete loss of remnant polarization (see $P_r(T)$ in Supplementary Fig. 2a). In this figure, a dramatic step between 100 °C ≤ T ≤ 105 °C is observed, which serves as direct evidence for the transition from the ferroelectric to the paraelectric phase. The temperature at which this phase transition occurs is referred to as the Curie temperature T_C. A T_C of about 105 °C—as derived for our ultraflexible ferroelectric transducer—is in good agreement with previously reported values for P(VDF:TrFE) layers with 70:30 mol%[2]. Noticeably, the Curie temperature for a non-poled layer is also located at 105 °C, as shown in Supplementary Fig. 2a; this implies that the intrinsic property of ferroelectricity, the existence of polar molecular crystallites with non-centric symmetry (as is the case in the all-trans conformation of PVDF), is not influenced by poling in our experiment. This result is in contrast to those in previous reports[3]. At temperatures below T_C, the permittivity of the poled layer $\varepsilon_{r,poled}$ is lower than that of the unpoled layer $\varepsilon_{r,unpoled}$ and, as shown in Supplementary Fig. 2b, it is nearly flat over a wide temperature range (i.e., from −100 to 90 °C). This phenomenon, i.e., the temperature insensitivity of $\varepsilon_{r,poled}$, is attributed to electrical poling that generally reduces disorder in the all-trans conformation of the ferroelectric phase, thereby resulting in a decrease in thermal fluctuations, and in turn, a decrease in permittivity[3].

Supplementary Note 2: Organic diode: Vertical Schottky setup

The structure of the vertical Schottky diode is displayed in Supplementary Fig. 12. A 50-nm Au electrode was thermally evaporated through a metal mask on the parylene substrate followed by PFBT-SAM (2,3,4,5,6-Pentafluorobenzenethiol, TCI chemicals) treatment. Prior to the SAM treatment, the samples were cleaned by oxygen plasma treatment at 100 W for 3 min. For the SAM treatment, the sample was immersed in a 10-mMol 2-propanol solution of PFBT for 3 h. After this immersion step, the samples were carefully rinsed with pure solvent and blown dry by nitrogen. Then, a 100-nm thick layer of DNTT was thermal evaporated at a rate of 0.3

Å s^{-1} and a pressure of 10^{-4} Pa. Finally, a top electrode was formed on the DNTT surface by thermally evaporating a 100-nm thick aluminium layer through a shadow mask.

The different injection barriers between the two different metals and the organic semiconductor are used for rectification. Because of their work functions, the Al electrode blocks the injection of holes and the Au electrode permits the injection of holes. Typical current/voltage characteristics of a vertical diode are plotted in Supplementary Fig. 12b, revealing a rectification ratio over 10^2 (between −5 V and +5 V) and a current density of about 0.3 A cm^{-2} at a forward voltage of +5 V. The overall diode performance could be strongly improved by introducing a PFBT-SAM between the Au and DNTT interface. The SAM treatment results in a decrease of the injection barrier (Supplementary Fig. 12a) and, as is often shown in literature[4-7], in an enhanced crystallinity and molecular packing of the organic semiconductor. The SAM-modified diodes feature a more than 30 times higher current density of 10 A cm^{-2} at +5 V, and four orders of magnitude higher rectifying ratio of > 10^6, high reverse breakdown voltages over −15 V and measured operation frequencies of a few megahertz (Supplementary Fig. 12c).

However, the SAM modification layer leads to an increase in the transition voltage from $V_T < 0.1$ V to $V_T >> 0.3$ V. This built-in voltage drop across the diode is a result of the relative difference between the Au and Al work function. The PFBT-SAM modification layer further enlarges this voltage gap by elevating the work function of the Au-electrode (Supplementary Fig. 12a)[4]. This effect deteriorates the rectifying performance of the diodes and makes it ineffective for energy harvesting applications because a part of the generated voltage is lost because of the large built-in voltage drop across the diodes. Another considerable disadvantage of the vertical diodes is that the overall device-to-device reproducibility and electrical stability are rather poor for both, SAM- and non-SAM-modified diodes, most probably caused by the oxidation processes near the aluminium/semiconductor interface.

As is obvious from Supplementary Table 3 the OTFT-based diodes with shorted drain-gates outperform the vertical diodes in terms of their low transition voltage, their better device-to-device reproducibility and their high electrical stability. These features are caused by the dielectric and interface properties of organic diodes in a shorted OTFT setup, where the charge carrier transport takes place at the smooth dielectric/semiconductor interface. This transport can be more easily controlled and optimised in shorted OTFT diodes than in lateral diodes, where the charge transport occurs vertically through the semiconducting layer and is prone to trapping.

Supplementary Note 3: Estimation of energy harnessing performance from biomechanical motion of multi-layer UFPTs

The energy delivered by our piezoelectric energy harvesting device strongly depends on the mounting position on the human body. When actuated by bending, it is important that a high stretching is introduced to the transducer. Therefore, places subject to a lot of muscle work, such as when the leg or arm muscles expand and contract, or where strong bending appears, like at the elbow joint or knee joint, could be ideal mounting spots for maximizing the harvesting. Another good position could be on/under the sole, which deforms a lot when walking. However, we admit that further experiments are necessary to investigate the UFPT harvesting performance on the different positions on the human body.

The amount of energy harvested from the motion of the elbow joint can be roughly estimated from our bending tests on a rail, as shown in Supplementary Fig. 15. Here the deformation of the rubber during bend and unbend positions is somehow similar to the elbow joint movement between flexion and extension position. The mean generated areal energy density E_{gen} for a single transducer layer was measured to be around 20 nJ cm^{-2} per cycle (bend and release). This was calculated by $E_{\mathrm{gen}} = E / (N \cdot A)$ where E is the stored energy in the capacitor (from Fig. 7d, C = 10 µF), N the number of bending cycles (450) and A (2.25 cm^2) the transducer area.

Stacking the ultraflexible transducers would be a promising approach for increasing the energy output as we demonstrated in this work. The generated charge level was either doubled for the two-layer stack or even tripled for the three-layer stack as compared to the maximum charge level measured for a single-layer UFPT under the same excitation conditions (Fig. 3d).

Placing a multiple layer stack of 25 transducers (yet amounting to a just 60 µm thin device) with an active area of 20 cm^2 on the elbow joint would generate about 10 µJ of energy per motion cycle (movement between flexion and extension position). Thus, for 100 movements / hour we estimate a harvested energy of 1 mJ per hour or 16 mJ per day if we assume an activity period of 16 hours (for storage capacitors with too small capacitance discharging effects might decrease these values to a certain extent).

Another good position to place the UFPT harvester would be the knee joint. Per day, an average person takes 2000-4000 steps in normal activities and over 10000 steps in sporting activities; walking would allow harvesting 20 to 100 mJ per day for one knee.

We believe that a feasible application scenario would be to continuously harvest and store biomechanical energy in an energy storage device until a certain charging level is reached. Then a pulse wave measurement will be triggered and the measured data will be stored in a data logger. Although in our opinion it is unlikely to achieve a continuous, uninterrupted recording

of vital parameters solely based on harvested energy, the charging period with a multiple stack of UFPTs can still be kept quite short (a few minutes), so as to allow for a periodic health tracking. The threshold charging level can be further adjusted to enable a wireless transfer of data to a computer or smartphone once or twice a day.

The update period critically depends on the power consumption of the electronic circuit. For this application, we would need a compact wireless electronic system operating in a special duty-cycle that allows ultra-low power consumption by remaining in a low/zero energy consumption state (sleep phase) most of the time and just consuming energy during measurements and communication in the active phase. We do not develop such a low power system but there are examples already presented in literature that can be suitable for this purpose. Ultra-low power wireless communication protocols with outstanding consumption figures of less than 300 nW and 1 mJ for the sleep and active phases, respectively were reported[8]. A comprehensive overview of different energy harvesting strategies is published in Singh et al.[9]. To summarize, from biomechanical motions we estimate that an energy amount of up to 200 mJ per day can be harvested, which may be sufficient to power a wireless electronic system in a duty-cycled operation and enables to transmit the measured pulse wave data several times a day (every 30-60 mins). These values are based on some partly rough assumptions, thus further research is necessary to investigate the real harvesting performance of UFPT's on human skin (maybe not only joints are good placing spots, muscle work (expansion and contraction) may also generate high signals…). In addition, a suitable wireless data processing and communication system has to be developed.

Supplementary Note 4: Calculation of the UFPT’s energy conversion efficiency

In similar previous studies (cf. Dagdevieren et al. and references therein)[10], the energy conversion efficiency of a transducer was calculated as the ratio of energy stored in the harvester to the total mechanical input energy, considering also the work performed on the underlying layer causing deformation of the harvester. However, the invested work to deform the underlying layer depends strongly on the layer’s mechanical properties, dimension, deformation shape etc. and varies among possible use cases. Therefore, we find it more appropriate to evaluate the ratio of output electric energy to the mechanical work performed on the UFPT, consisting of the piezoelectric layer and the substrate. The overall efficiency can then be written as

$$\eta = \eta^{\mathrm{el}} \cdot \eta^{\mathrm{m}} \quad (1)$$

with

$$\eta^{\mathrm{m}} = \frac{W_{\mathrm{t}}^{\mathrm{m}}}{W_{\mathrm{sub}}^{\mathrm{m}} + W_{\mathrm{t}}^{\mathrm{m}}} \tag{2}$$

and

$$\eta^{\mathrm{el}} = \frac{W_{\mathrm{t}}^{\mathrm{el}}}{W_{\mathrm{t}}^{\mathrm{m}}} \tag{3}$$

Here, $W_{\mathrm{t}}^{\mathrm{el}}$ and $W_{\mathrm{t}}^{\mathrm{m}}$ denote the stored electrical energy and mechanical strain energy in the piezoelectric material, respectively, and $W_{\mathrm{sub}}^{\mathrm{m}}$ is the mechanical strain energy stored in the passive substrate.

Since the stored strain energy during deformation is not accessible through experiment, we applied a three-dimensional FEM simulation to numerically derive the respective energy quantities for the case of bending on a rubber layer (mode B) and also compared them with the predictions of an analytical model.

Supplementary Note 4.1: FEM simulation

The FEM model of the transducer was the same as for the transversal load simulation, see Methods, Chapter 7 and Supplementary Table 2. The relative permittivity, ε_{r}, of the piezoelectric layer was taken to be 8.5. The transducer was placed centrally on top of a 10 × 10 cm^2 rubber sheet with 2 mm thickness (with a Young's modulus of 1.45 MPa and a Poisson ratio of 0.49) without allowing for friction or sliding. Clamping conditions were applied on both ends of the rubber over an area of 2 cm × 1 cm mimicking the metal clamps in the experiment. Symmetry was employed in the *xz*- and *yz*-planes normal to the rubber surface to reduce the computational complexity by a factor of ≈ 4. The lateral displacement of the clamped region causing upward displacement and convex bending of the rubber was simulated with displacement values used during the experiment. Supplementary Fig. 19a depicts the model at maximum bending (cf. Supplementary Fig. 16a, photograph at maximum bending). To calculate the output currents and electric energy, the reduced transducer element was virtually either short-circuited or connected to a load resistance of $R_{\mathrm{L,sym}} = 4 \cdot 2.5\ M\Omega$, which corresponds to the optimum load under experimental condition of 2.5 MΩ (cf. Supplementary Fig. 16b), where the factor 4 is due to the symmetry of the model. A time study step was performed with a triangular displacement profile over a period of 0.5 s (onset at t = 0.25 s), which corresponds to the average excitation frequency in the experiment (2 Hz). The time plot in Supplementary Fig. 19b shows the currents at short-circuit and load condition, respectively, as well as the power dissipated by the load normalized by the transducer volume for direct comparison with the experimental values in Supplementary Fig. 16b. The volumetric peak

power density amounts to ≈1.1 mW cm^{-3} and is in excellent agreement with the experimental value (cf. Table 1). The electrical energy per area for a single load cycle was calculated by a time integration of the power dissipated over the load resistance and amounted to 72 nJ cm^{-2}, which is 3.6 times higher than in the experiment. This discrepancy in total energy output per cycle might be due to a different load profile in the experiment or non-perfect adhesion of the UFPT on the substrate in the experiment causing perhaps a transducer displacement or slipping in the initial actuation cycle, which is difficult to account for in the simulation. The performed mechanical work was numerically calculated for the piezoelectric and substrate layer, respectively, as (Einstein summation convention applied)

$$W^{\mathrm{m}} = \iint \sigma_i \, d\epsilon_i \, dV \tag{4}$$

Static study steps were performed with varying substrate thicknesses D_{sub} to derive a trend of the energy conversion coefficients η^{m} and η^{el} at full bending of the rubber layer. The internally stored electric energy at full bending, $W^{\mathrm{el}} = \frac{\Delta Q_{\mathrm{t}}}{2\, C_{\mathrm{t}}}$, with displacement charge ΔQ_{t} and the transducer's capacitance C_{t}, was used to derive η^{el} in the static case.

Supplementary Note 4.2: Analytical model

To derive the theoretical energy conversion efficiency of the piezoelectric P(VDF-TrFE), $\eta^{\mathrm{el}}_{\mathrm{th}}$, we consider the case of uniaxial in-plane stretching with clamping applied to the lateral direction. This shall mimic the situation of our UFPT being adhered to a deforming surface (in the *xy*-plane) and undergoing a unidirectional stretching (*x*-direction) due to bending of the surface (bending axis pointing in *y*-direction). The piezoelectric constitutive equations in pseudovector form are[11]

$$\epsilon_p = s^E_{pq}\, \sigma_q + d_{ip}\, E_i \tag{5}$$

$$D_i = d_{iq}\, \sigma_q + \varepsilon\, E_i \tag{6}$$

where $\boldsymbol{\epsilon}$ and $\boldsymbol{\sigma}$ are the strain and stress pseudovectors, $\mathbf{s}^E$ is the compliance matrix at constant field, $\varepsilon = \varepsilon_0 \varepsilon_{\mathrm{r}}$ is the permittivity, **D** the electric displacement, **E** the electric field and *d* the matrix with piezoelectric coefficients (i=1, 2, 3 and $p, q = 1 \ldots 6$). For the case of uniaxial loading in the *x*- or *1*-direction, the clamping condition leads to the following boundary conditions: $\epsilon_2 = 0, \sigma_3 = 0$. In addition, no shear strains/stresses shall appear. The polarization points to the 3-direction. With electrodes applied on the top and bottom, only $E_3 \neq 0$. Applied to Supplementary Equation (5), we obtain:

$$\epsilon_1 = s_{11}^E\sigma_1 + s_{12}^E\sigma_2 + d_{31}E_3 \quad (7)$$

$$\epsilon_2 = s_{21}^E\sigma_1 + s_{22}^E\sigma_2 + d_{32}E_3 = 0 \quad (8)$$

The performed mechanical work per volume during deformation of the piezoelectric layer, w^{m}, is (where volumetric changes due to bending are neglected)

$$w^{\mathrm{m}} = \int \sigma_1 d\epsilon_1 \quad (9)$$

Applying Supplementary Equation (7) and (8) for constant voltage condition ($dE_3 = 0$) yields

$$w^{\mathrm{m}} = \left(s_{11}^E - \frac{s_{12}^{E^2}}{s_{22}^E}\right)\cdot\frac{\Delta\sigma_1^2}{2} \quad (10)$$

The electrical field generated during deformation can be obtained from Supplementary Equation (6) at open-circuit condition ($D_3 = 0$) and amounts to

$$E_3 = -\frac{\left(d_{31} - d_{32}\frac{s_{12}^E}{s_{22}^E}\right)}{\left(\varepsilon - \frac{d_{32}^2}{s_{22}^E}\right)}\cdot\Delta\sigma_1 \quad (11)$$

When connected to a perfect load, the available electrical energy per volume, w^{el}, during release is then

$$w^{\mathrm{el}} = \int E_3 dD_3 = \int_{\Delta\sigma_1}^{0} E_3\left(d_{31} - d_{32}\frac{s_{12}^E}{s_{22}^E}\right)d\sigma_1 = \frac{\left(d_{31} - d_{32}\frac{s_{12}^E}{s_{22}^E}\right)^2}{\left(\varepsilon - \frac{d_{32}^2}{s_{22}^E}\right)}\cdot\frac{\Delta\sigma_1^2}{2} \quad (12)$$

Thus, for the energy conversion ratio we get:

$$\eta_{\mathrm{th}}^{\mathrm{el}} = \frac{w^{\mathrm{el}}}{w^{\mathrm{m}}} = \frac{\left(d_{31} - d_{32}\frac{s_{12}^E}{s_{22}^E}\right)^2}{\left(\varepsilon - \frac{d_{32}^2}{s_{22}^E}\right)\left(s_{11}^E - \frac{s_{12}^{E^2}}{s_{22}^E}\right)} \quad (13)$$

Using the isotropic model for P(VDF-TrFE) (see Supplementary Note 7) and applying the dimension model for its piezoelectricity (with piezoconstants $e_{33} = -P_{\mathrm{r}}$, $e_{31} = e_{32} = 0$)[12], the piezoelectric coefficients d_{ij} can be obtained from the compliance matrix and the remnant polarization P_{r} as

$$d_{3j} = -P_{\mathrm{r}}\, s_{3j}^E \quad (14)$$

With this, Supplementary Equation (13) becomes

$$\eta_{\mathrm{th}}^{\mathrm{el}} = \frac{P_{\mathrm{r}}^2}{\varepsilon_0\varepsilon_{\mathrm{r}} - \frac{\nu_{\mathrm{t}}^2}{E_{\mathrm{t}}}P_{\mathrm{r}}^2}\cdot\frac{\nu_{\mathrm{t}}^2}{E_{\mathrm{t}}}\frac{(1+\nu_{\mathrm{t}})}{(1-\nu_{\mathrm{t}})} \quad (15)$$

where E_{t} is the Young's modulus and ν_{t} is the Poisson ratio of the P(VDF-TrFE).

For the sample used at the bending test (see Supplementary Table 2) this gives $\eta_{\mathrm{th}}^{\mathrm{el}}$ = 0.36 %, corresponding to a mechanical coupling coefficient $k_{31}^{\mathrm{w}} = \sqrt{\eta_{\mathrm{th}}^{\mathrm{el}}} = 0.06$ [11].

The mechanical energy efficiency, which we define here as $\eta^{\mathrm{m}} = \frac{W_{\mathrm{t}}^{\mathrm{m}}}{W_{\mathrm{sub}}^{\mathrm{m}} + W_{\mathrm{t}}^{\mathrm{m}}}$, (cf. Equation (3)) can be derived as follows. Using Supplementary Equation (9), the total mechanical work performed during (elastic) bending of a layer at radial position $z = z_{\mathrm{l}}$, length l, and width b is

$$W^{\mathrm{m}} = \int w^{\mathrm{m}}\, dV = b\, l \int_{z_{\mathrm{l}}}^{z_{\mathrm{l}}+t} \sigma_1\, d\epsilon_1\, dz \qquad (16)$$

The strain introduced by bending at a radius R is $\epsilon_1(z) = (z - z_{\mathrm{N}})/R$, where z_{N} is the radial z-position of the neutral mechanical plane (NMP). Using $\sigma_1 = E_{\mathrm{eff}}\, \epsilon_1$ we obtain

$$W^{\mathrm{m}} = \frac{b\, l}{2}\; t\, E_{\mathrm{eff}} \frac{d^2}{R^2} \left(1 + \frac{t}{d} + \frac{t^2}{3\, d^2}\right) \qquad (17)$$

with $d = z_{\mathrm{l}} - z_{\mathrm{N}}$ being the radial distance to the NMP. The effective Young's modulus, E_{eff}, depends again on the clamping condition and material property. For an isotropic material and with the same clamping condition as above ($\epsilon_2 = 0$), one gets $E_{\mathrm{eff}} = E/(1 - \nu^2)$. In the case of $t \ll d$, Supplementary Equation (17) reduces to

$$W^{\mathrm{m}} = \frac{b\, l}{2} \frac{t\, E}{1-\nu^2} \frac{d^2}{R^2} \qquad (18)$$

Next, we can derive Supplementary Equation (19) as the ratio of mechanical work stored in the piezoelectric layer (thickness D_{t}) and the substrate layer (thickness D_{sub}) assuming both have the same footprint (i.e. $l = L_{\mathrm{t}} = L_{\mathrm{sub}}$, $b = b_{\mathrm{t}} = b_{\mathrm{sub}}$):

$$r_{\mathrm{th}} = \frac{W_{\mathrm{t}}^{\mathrm{m}}}{W_{\mathrm{sub}}^{\mathrm{m}}} = \frac{D_{\mathrm{t}}}{D_{\mathrm{sub}}} \cdot \frac{E_{\mathrm{t}}}{E_{\mathrm{sub}}} \cdot \frac{1-\nu_{\mathrm{sub}}^2}{1-\nu_{\mathrm{t}}^2} \qquad (19)$$

The theoretical mechanical energy efficiency is then

$$\eta_{\mathrm{th}}^{\mathrm{m}} = \frac{r_{\mathrm{th}}}{(1+r_{\mathrm{th}})} \qquad (20)$$

Obviously, it scales with the ratio of piezoelectric layer thickness vs. substrate thickness, $D_{\mathrm{t}}/D_{\mathrm{sub}}$. As can be seen in Supplementary Fig. 20a, the mechanical energy efficiency as predicted by the model is also in excellent agreement with the numerically calculated values based on the 3D FEM simulation. For the presented UFPT with an only 1 µm thin substrate, it amounts to 52 %. The overall theoretical energy efficiency for the presented UFPT is $\eta_{\mathrm{th}} = \eta_{\mathrm{th}}^{\mathrm{el}} \cdot \eta_{\mathrm{th}}^{\mathrm{m}} = 0.185$ %. From the FEM we obtain $\eta = 0.139$ %. The theoretical values are slightly larger compared to the values calculated with FEM (Supplementary Fig. 20b). In the FEM simulation the clamping conditions and bending of the transducer are closer to the real situation and thus more complex, which obviously reduces the effective electromechanical coupling in the piezoelectric layer (η^{el}).

Supplementary Figures

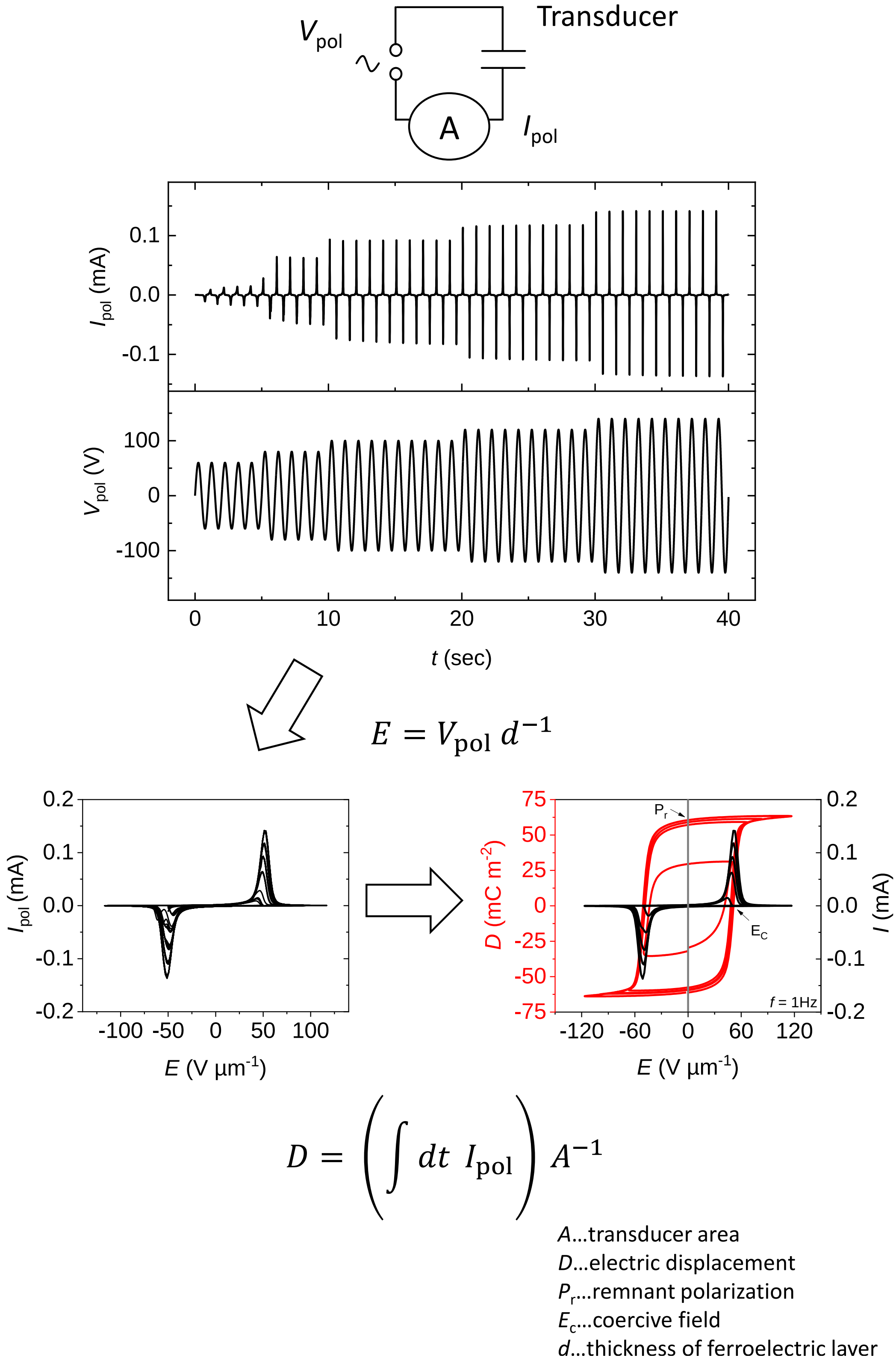

Supplementary Fig. 1 | Schematic procedure for 'poling' of a ferroelectric transducer.

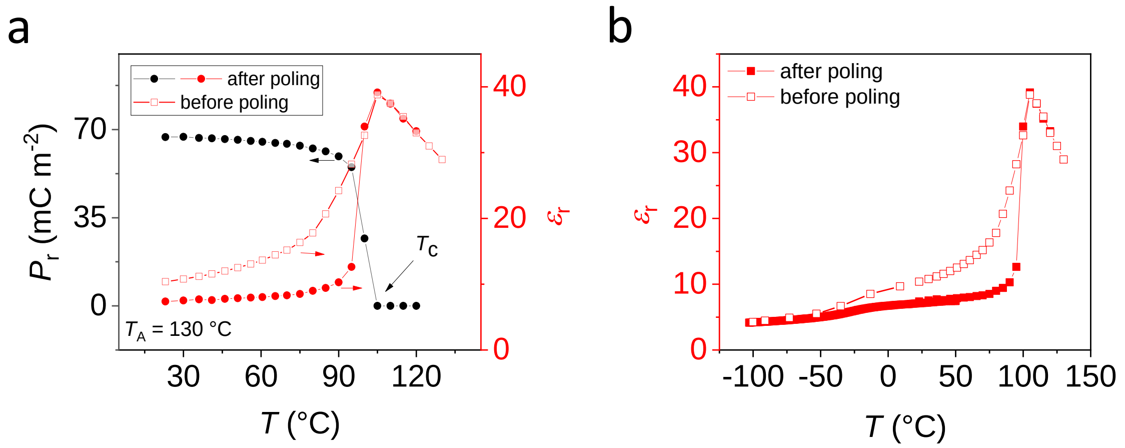

Supplementary Fig. 2 | Ferroelectric-to-paraelectric phase transition. a Temperature dependence of P_r and the relative permittivity ε_r associated with the ferroelectric-paraelectric phase transition in a 1.5-µm thin P(VDF:TrFE)$_{70:30}$ copolymer layer that was annealed at 130 °C. The Curie temperature (T_C) is about 105 °C. At T_C, the ferroelectric-to-paraelectric phase transition is observed. The relative permittivity was measured before and after electrical poling of the layer. **b** Temperature dependence of the relative permittivity ε_r of a 1.5-µm thin P(VDF:TrFE)$_{70:30}$ copolymer annealed at 130 °C measured in the range −100 °C to 125 °C. The relative permittivity was measured before and after electrical poling of the ferroelectric layer at 1 kHz.

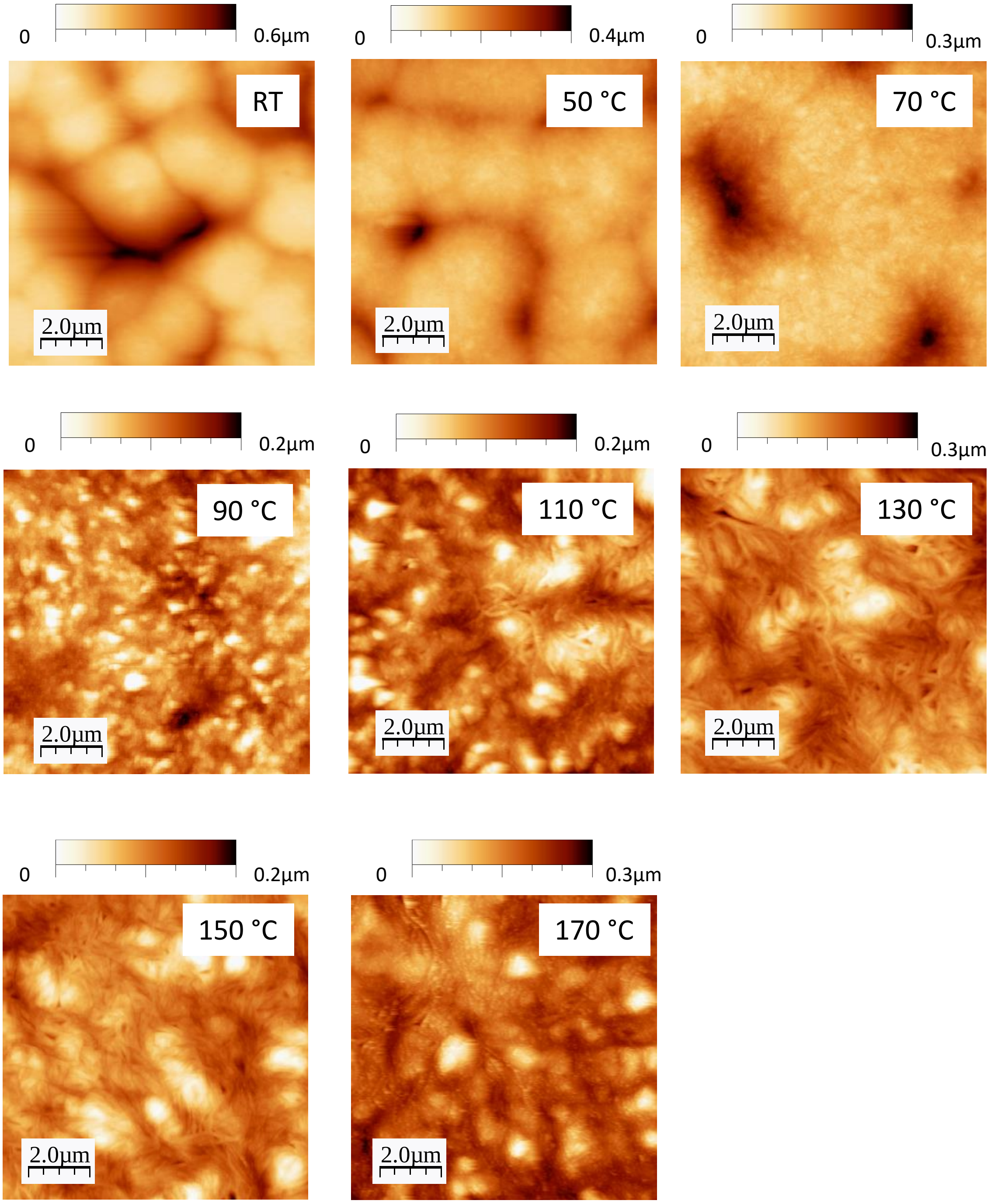

Supplementary Fig. 3a | AFM-images of P(VDF:TrFE) films for different annealing temperatures. AFM topography images (tapping mode, 10 µm × 10 µm scan size) of ~ 1.4 µm thin P(VDF:TrFE)$_{70:30}$ layers annealed between RT and 170 °C. (The colour scale shows the height.)

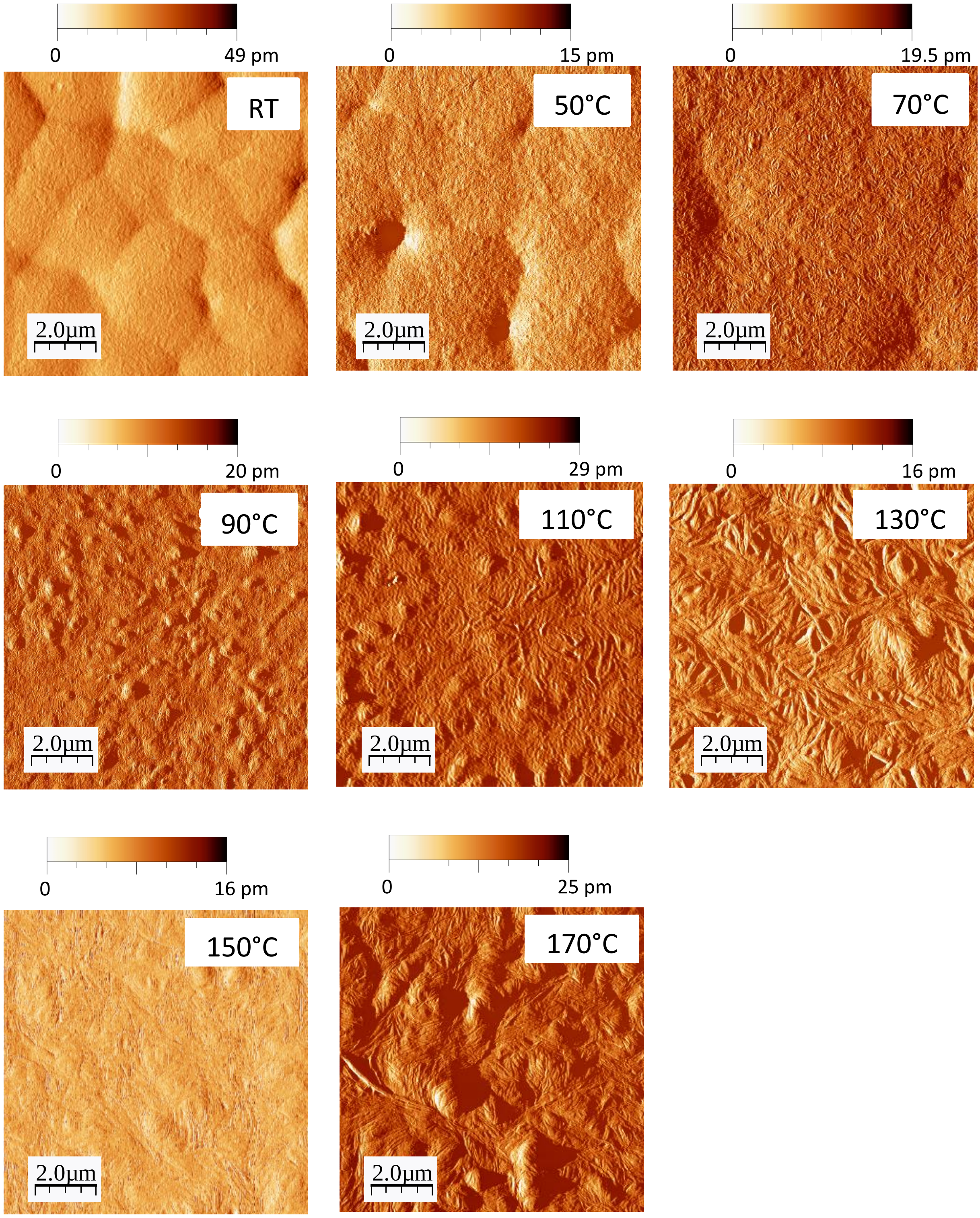

Supplementary Fig. 3b | AFM-images of P(VDF:TrFE) films for different annealing temperatures. Characteristic non-contact (NC) topographical atomic force microscopy images for different annealing temperatures T_A of spin-coated P(VDF:TrFE)$_{70:30}$ thin films (thickness ≈ 1.4 μm). (The colour scale shows the amplitude shift.)

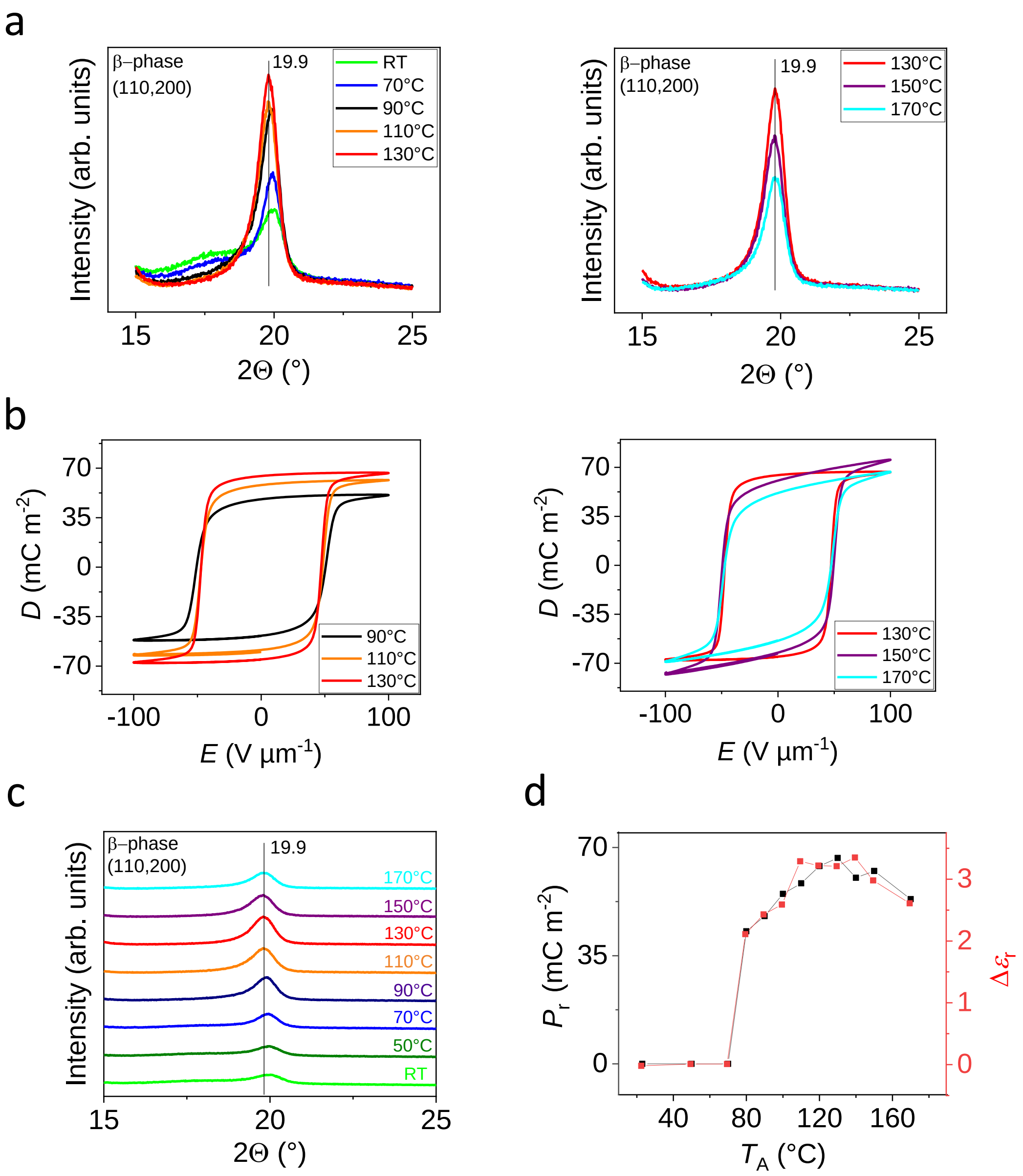

Supplementary Fig. 4 | XRD-pattern of P(VDF:TrFE) films for different annealing temperatures. a, **c** XRD-measurements of the P(VDF:TrFE)$_{70:30}$ layer for different annealing temperatures T_A. **b** Poling curves for different T_A after spin coating of the ferroelectric polymer. **d** Change in the relative permittivity $\Delta\varepsilon_r = \varepsilon_{r,\text{ before poling}} - \varepsilon_{r,\text{ after poling}}$ due to electrical poling as a function of various annealing temperatures T_A.

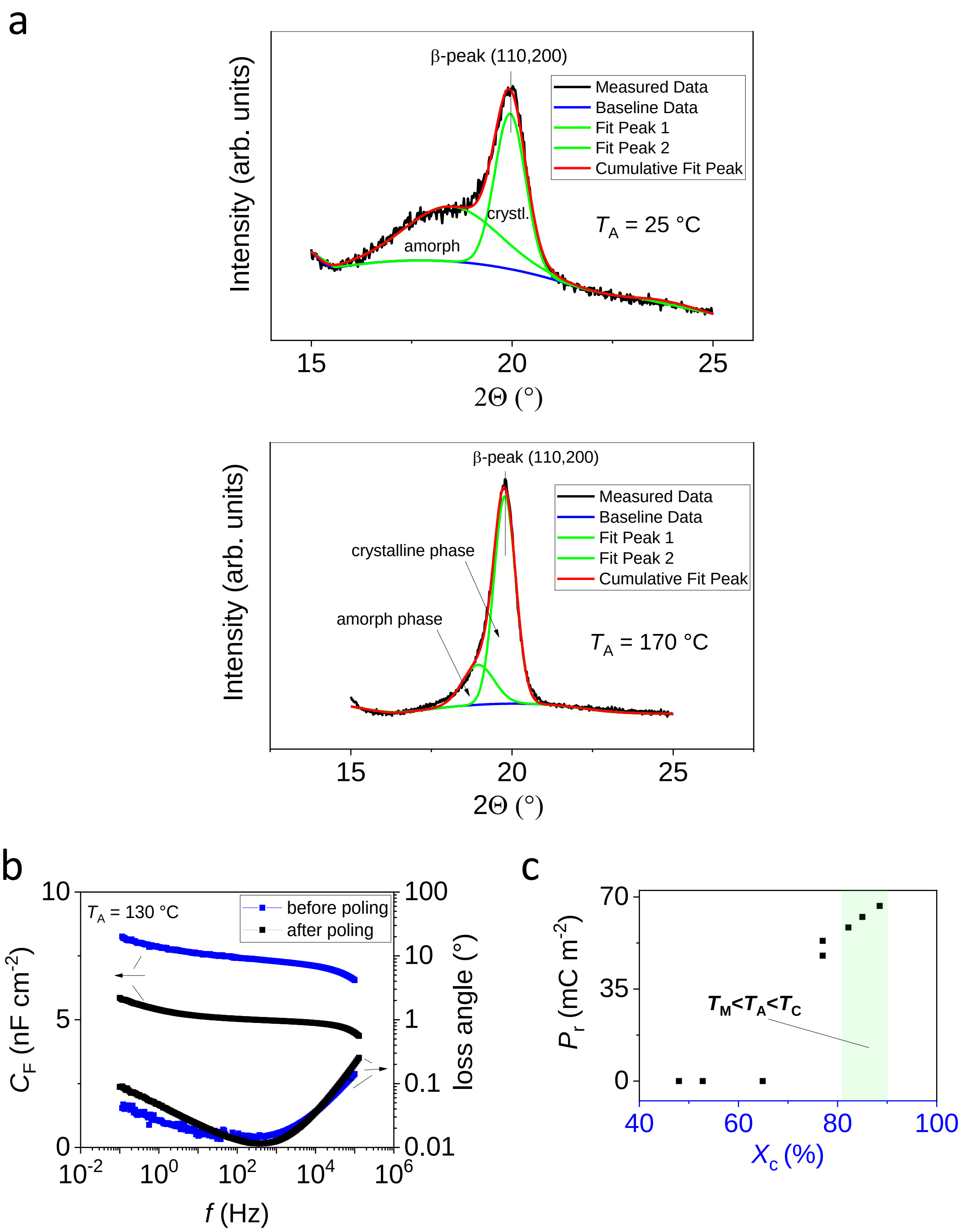

Supplementary Fig. 5 | Determination of crystallinity X_c. a Diffraction pattern and fitting plots to determine the degree of "apparent" crystallinity X_c for a 1.4-µm thick P(VDF:TrFE) layer annealed at RT and 170 °C. The XRD-signal around 2Θ: ~ 20° can be fitted by the superposition of two Gaussian functions: one attributed to the (110) and the (200) reflection of the copolymer's *β*-phase at 2Θ = ~ 19.9°, and the other to the amorphous phase. The fit yields the integrated peak areas and a refinement of the peak positions from which X_c can be determined. **b** Impedance measurements of a ferroelectric copolymer transducer (≈ 1 cm^2 area), annealed at 130 °C, show the frequency dependence of the capacitance C_F and the loss angle before and after electrical poling. **c** Remnant polarization P_r as a function of the crystallinity X_c of the ferroelectric co-polymer layer. The highest polarization values were observed for crystallinity levels between 80 and 90% achieved for annealing temperatures T_A between the melting temperature T_M and the Curie temperature T_C.

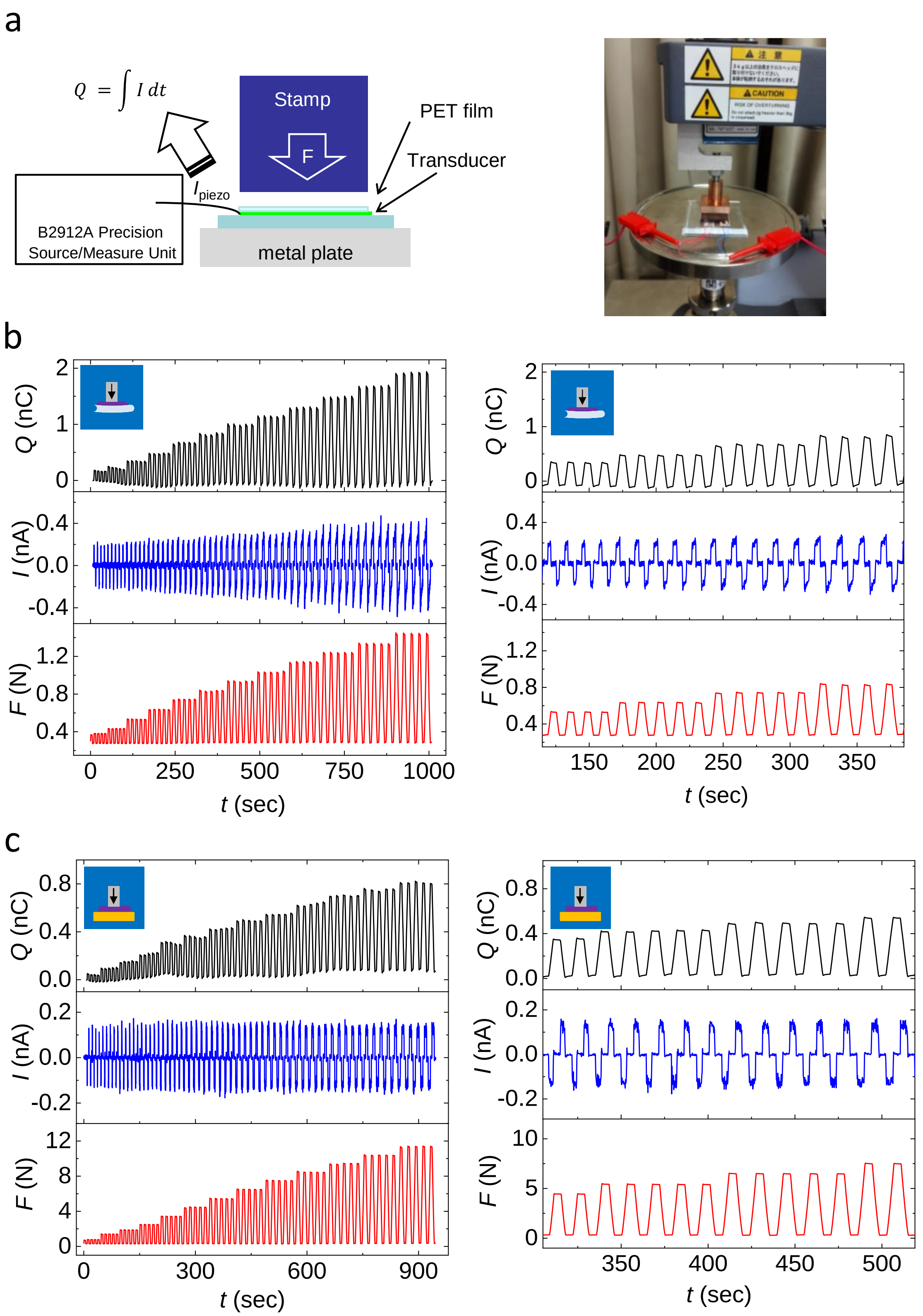

Supplementary Fig. 6 | Transversal load tests on various substrates. a Schematic image and photograph of the transversal load test setup (contact area: 2 cm^2, transducer area 2.25 cm^2). **b-c** Current response *I* and calculated charge response *Q* of one transducer layer attached (**b**) on silicone rubber for loads between 0.25 and 1.25 N and (**c**) on the glass for loads between 0.25 N and 10.25 N.

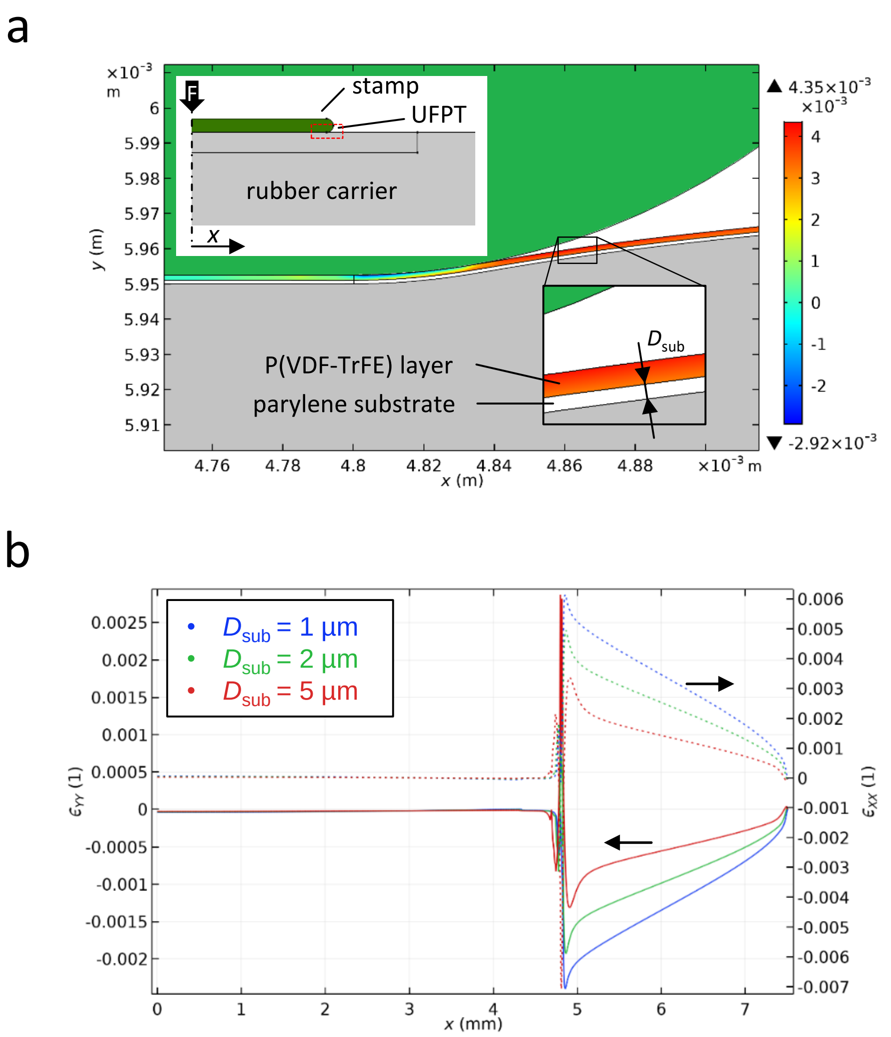

Supplementary Fig. 7 | FEM simulation of the transversal load test on elastic rubber carrier. a Calculated volumetric strain (colour scale) in the piezoelectric layer of the UFPT near the stamp edge at $F = 4$ N ($\varepsilon > 0$: tensile strain, $\varepsilon < 0$: compressive strain). The inset shows the 2D model with a symmetry plane at $x = 0$ m. The elastic rubber carrier has a thickness of 6 mm and the parylene substrate thickness is 1 µm, in accordance with the experiment depicted in Fig. 3c (Supplementary Table 2). **b** Local transversal and longitudinal strain components ϵ_{YY} (solid lines) and ϵ_{XX} (dotted lines), respectively, in the piezoelectric layer for three different thickness values D_{sub} of the UFPT substrate (here parylene). Strong deformation occurs near the edge of the stamp ($x \sim 5$ mm) because of the elastic underground, with pronounced longitudinal tensile strain and transversal compressive strain outside the stamp area. The locally induced piezoelectric polarization P_{piezo} is directly related to the transversal strain via the remnant polarization, $P_{\mathrm{piezo}} = -e_{YY} \cdot P_{\mathrm{r}}$. Obviously, the thinner the substrate layer of the UFPT is, the more the deformation of the rubber transfers into the ferroelectric layer causing a stronger piezoelectric response.

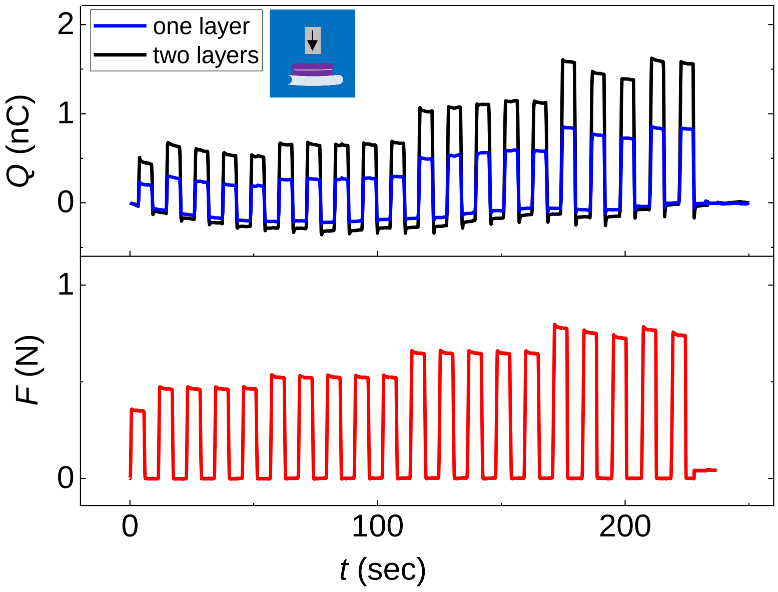

Supplementary Fig. 8 | Transversal load tests: Multiple layers. Charge response upon transversal load of one and two transducer layers attached on a 6-mm-thick silicone rubber carrier.

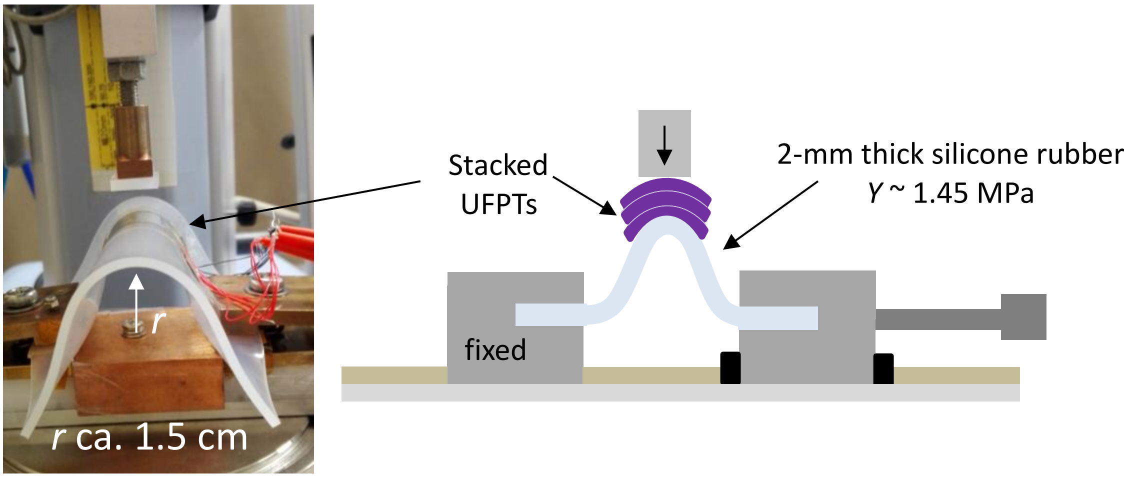

Supplementary Fig. 9 | Transversal load tests: Multiple layers. Photograph and schematics of the transversal load setup for UFPTs films attached on a pre-bent silicone rubber carrier.

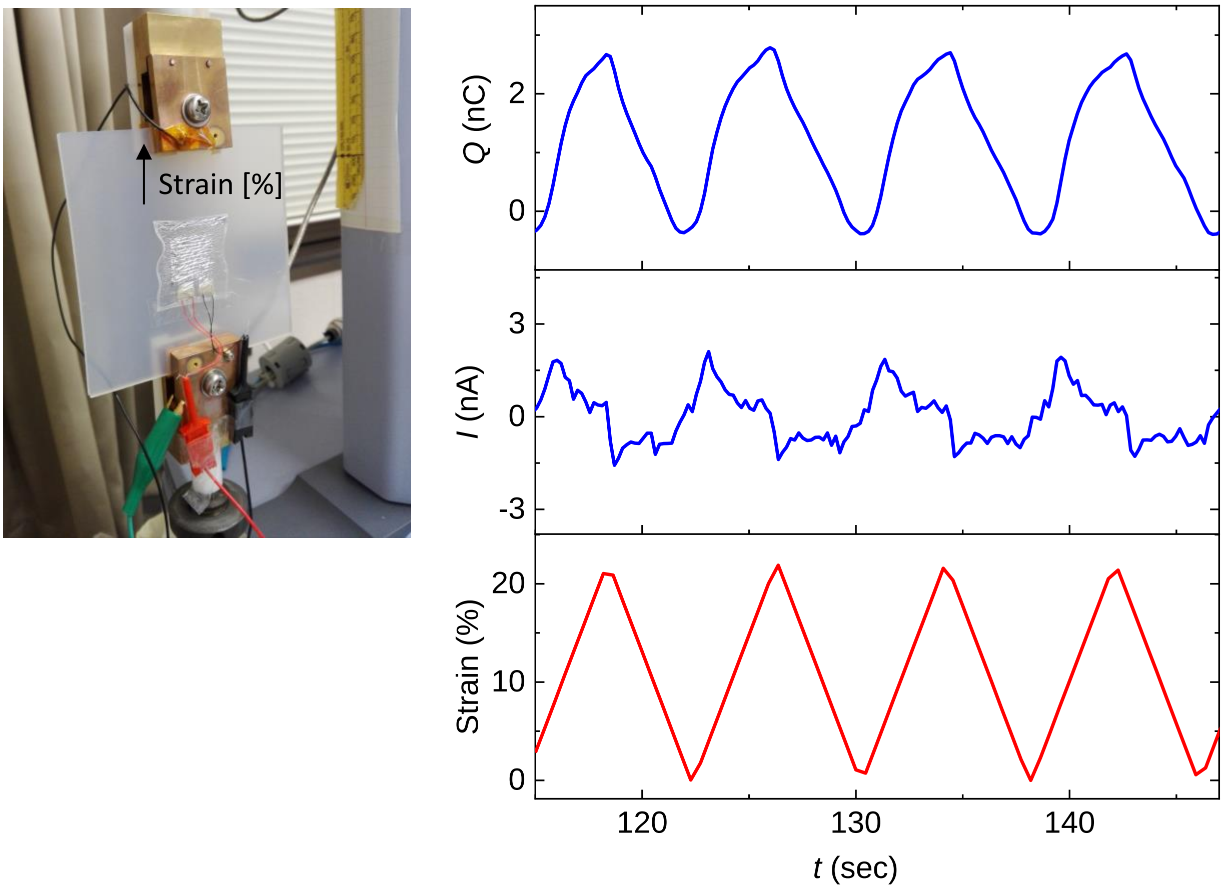

Supplementary Fig. 10 | Strain tests. Photo of the strain measurement setup showing an ultraflexible transducer attached to a pre-stretched 2-mm thin silicone rubber carrier. Current and charge response are plotted for strains up to 20 %.

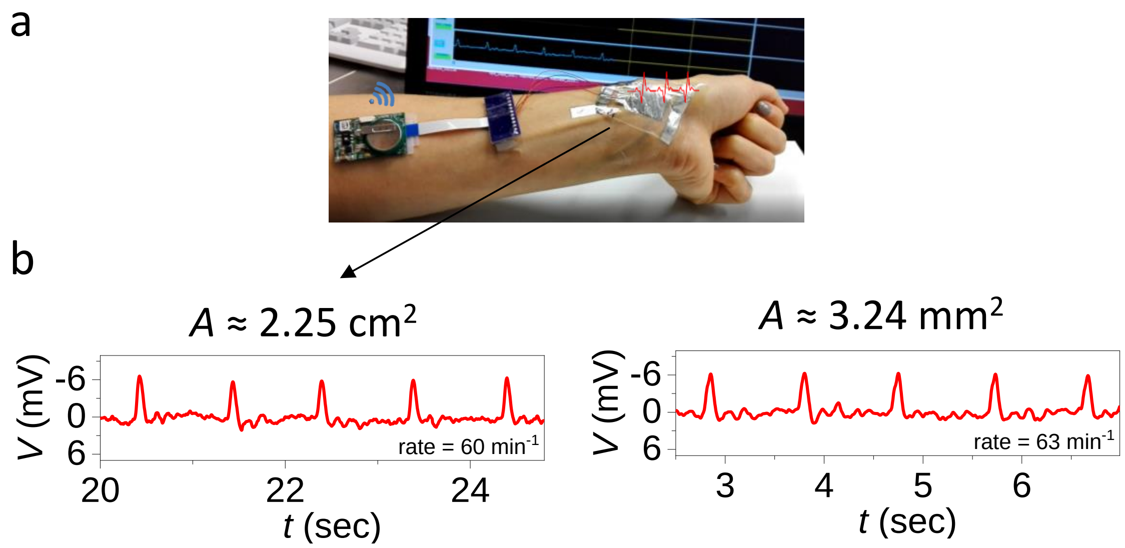

Supplementary Fig. 11 | Pulse wave measurements on the wrist. a Photograph of pulse wave measurement with an ultra-flexible piezoelectric transducer conformable attached on the wrist and connected to a wireless module with a 3.3 MΩ resistor parallel. **b** The human pulse wave associated with the flow of blood through near-surface arteries was monitored by a patch with an active sensing area of 2.25 cm^2 (left) and 3.24 mm^2 (right), respectively. A pulse rate of 60 min^{-1} could be extracted for the large sensor area and 63 min^{-1} for the small sensor area.

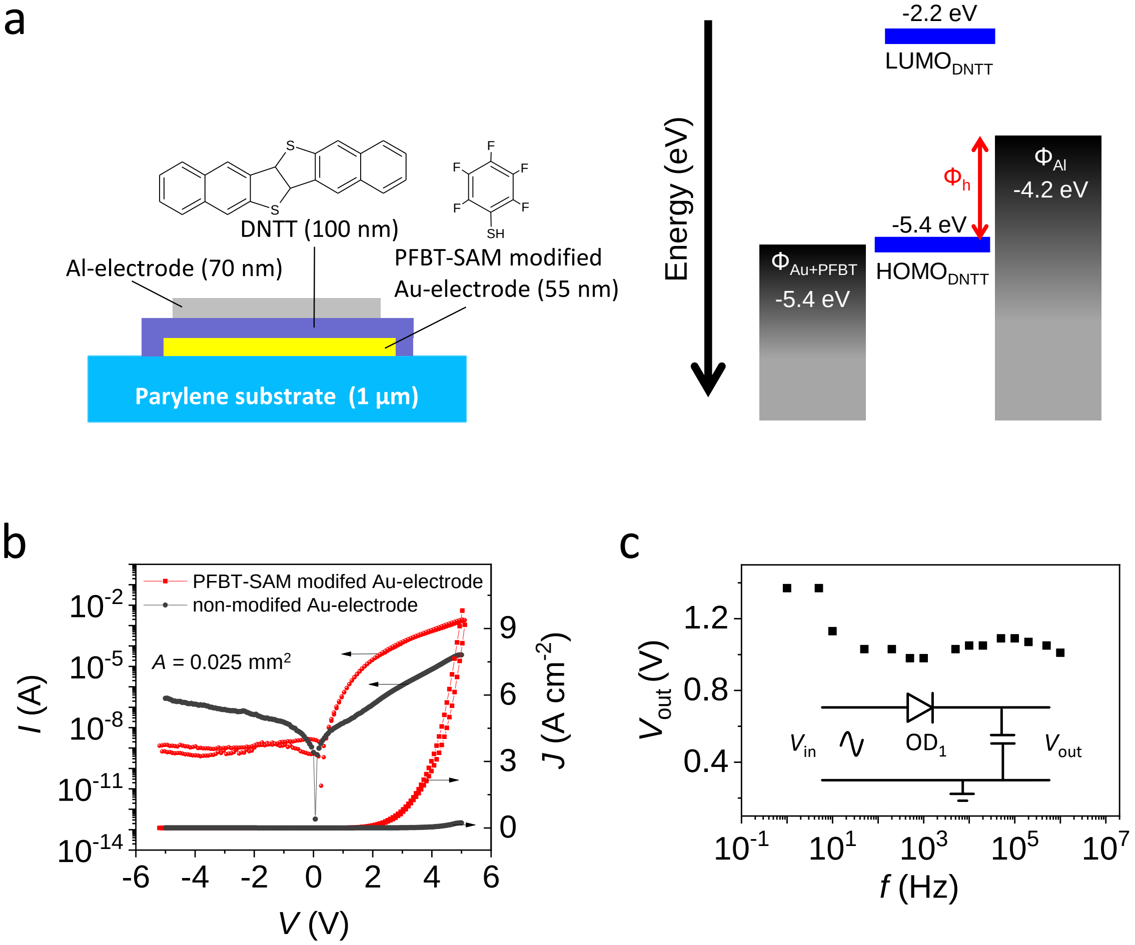

Supplementary Fig. 12 | Organic diode: Vertical Schottky setup. a Image of a vertical organic diode stack, and schematic of approximate energy levels for the different materials in the device stack; the HOMO/LUMO levels for DNTT are from the work of Yamamoto et al.[13]. The work functions of the modified and non-modified Au and Al electrodes are from Kuzumoto et al.[4] and Kumar et al.[14], respectively. **b** *I*/*V* and *J*/*V* curve of a vertical Schottky diode with and without PFBT SAM-modified electrodes. **c** DC-output voltage vs. frequency of an organic diode (OD_1) in a half-wave rectifier configuration (C = 100 nF).

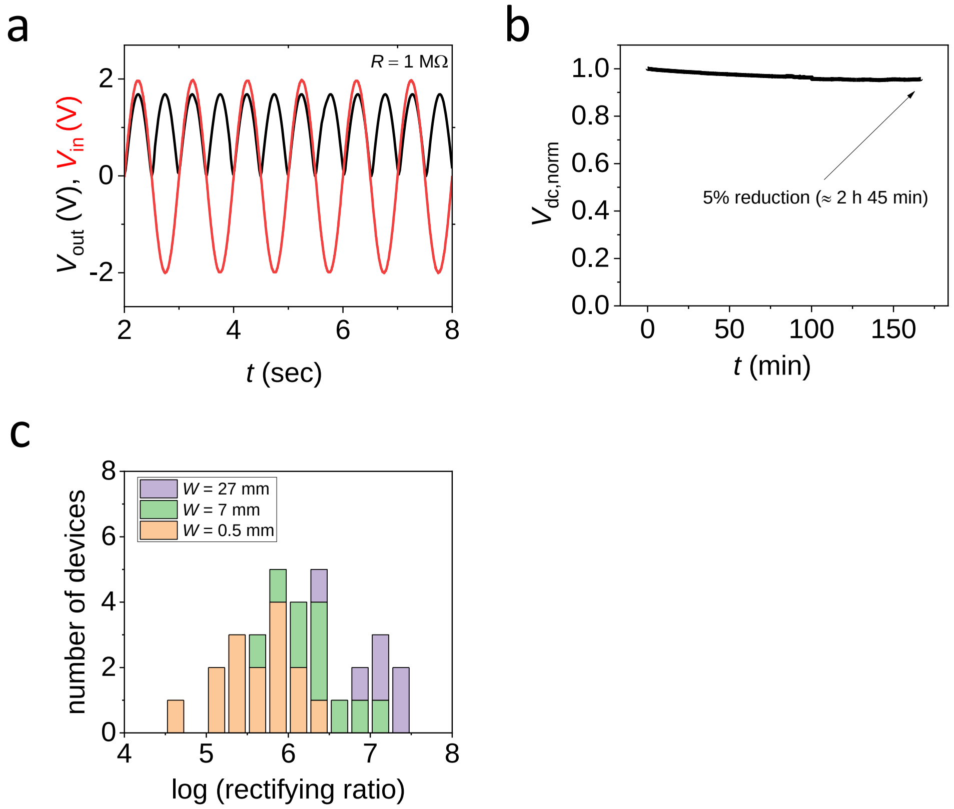

Supplementary Fig 13 | OTFT-based organic diodes. a AC input voltage V_{in} (red) and DC output voltage V_{out} (black) after rectification with an OTFT-based full wave rectifier circuit (OFWR) and connection to a 1 MΩ resistor. The OTFTs in the OFWR circuit have an AlO_x + SAM gate dielectric, a DNTT semiconductor and a W/L = 7 mm/ 12 µm. **b** Time dependence of the normalized output voltage $V_{dc,norm}$ of the OFWR for $V_{in} = 3$ V $\sin(\omega \cdot t)$, $f = 0.1$ Hz, and C = 10 µF. After 2 h and 45 min continuous operation, $V_{dc,norm}$ was reduced by not more than 5 %. **c** Histogram of the rectifying ratios of 31 OTFT-based organic diodes with a channel length of 12 µm and varying channel widths – 15 diodes have a channel width of 0.5 mm (orange), ten diodes have a channel width of 7 mm (green), and six diodes have a channel width of 27 mm (purple).

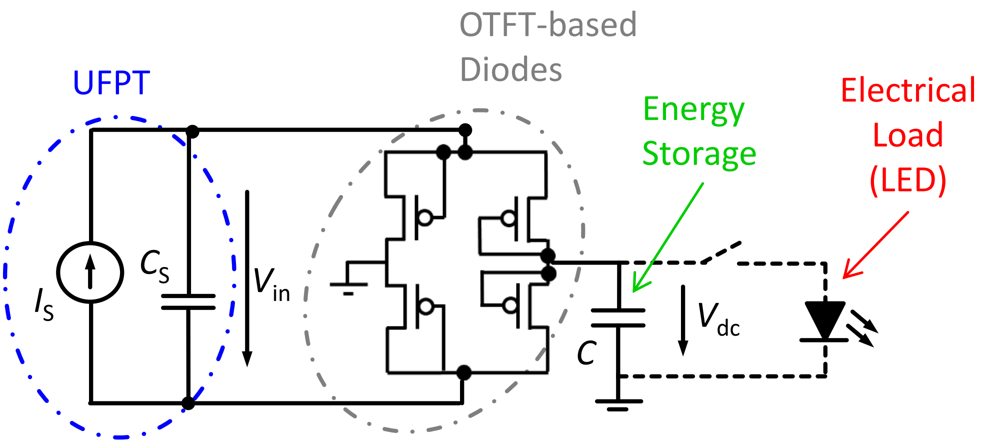

Supplementary Fig. 14 | Ultraflexible energy harvesting device (UEHD). Equivalent circuit diagram of the ultraflexible energy harvesting device comprising UFPT, full wave diode-based rectifier circuit, thin film capacitor (*C*) and an LED as the electrical load.

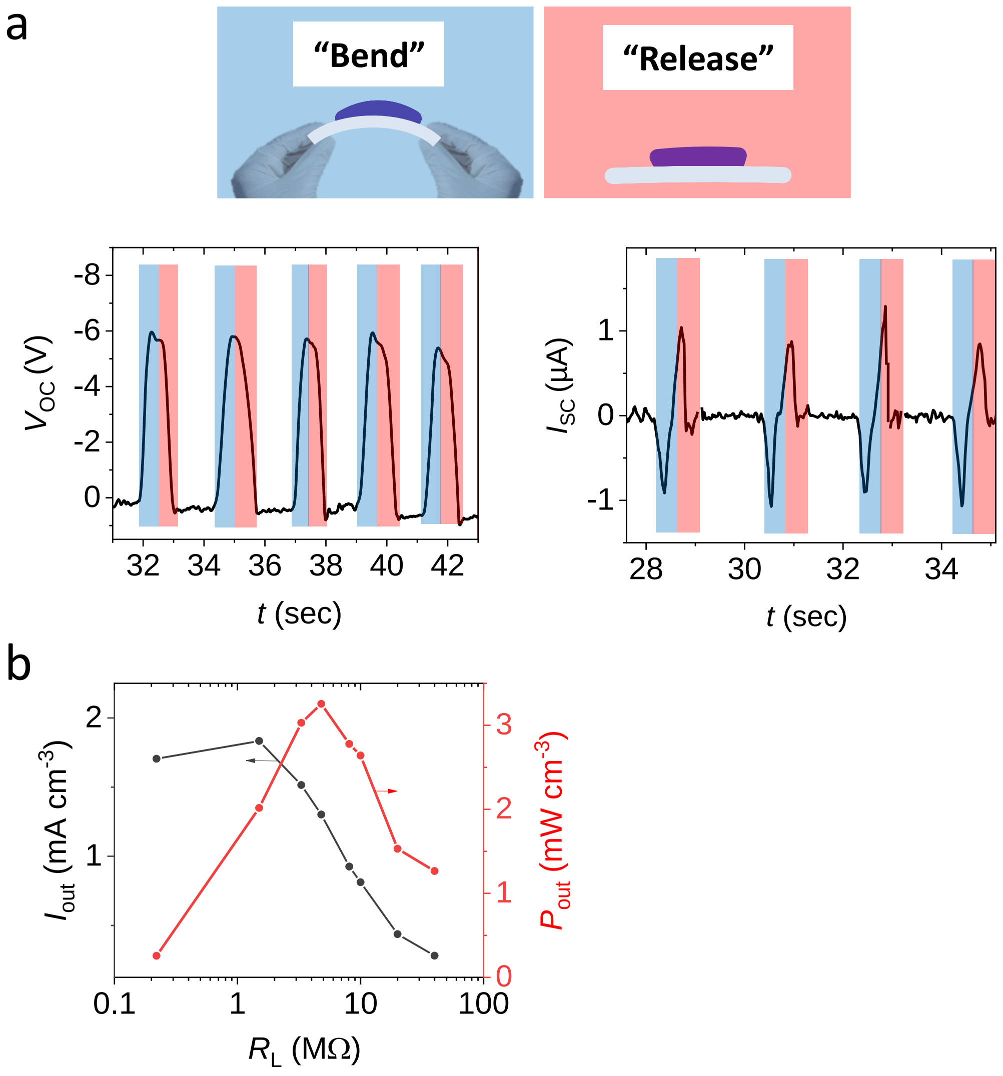

Supplementary Fig. 15 | Energy harvesting: Energy output by periodic manual bending of one UFPT (mode A). a Time evolution of the electrical energy output of an UFPT (open circuit voltage V_{oc} and short-circuit current I_{sc}) for manual periodic bending at ~ 2 Hz, blue phase is for bending and red phase is for releasing. **b** The current density I_{out} as well as the corresponding output power density P_{out} are plotted as a function of load resistance R_L for such an excitation scheme.

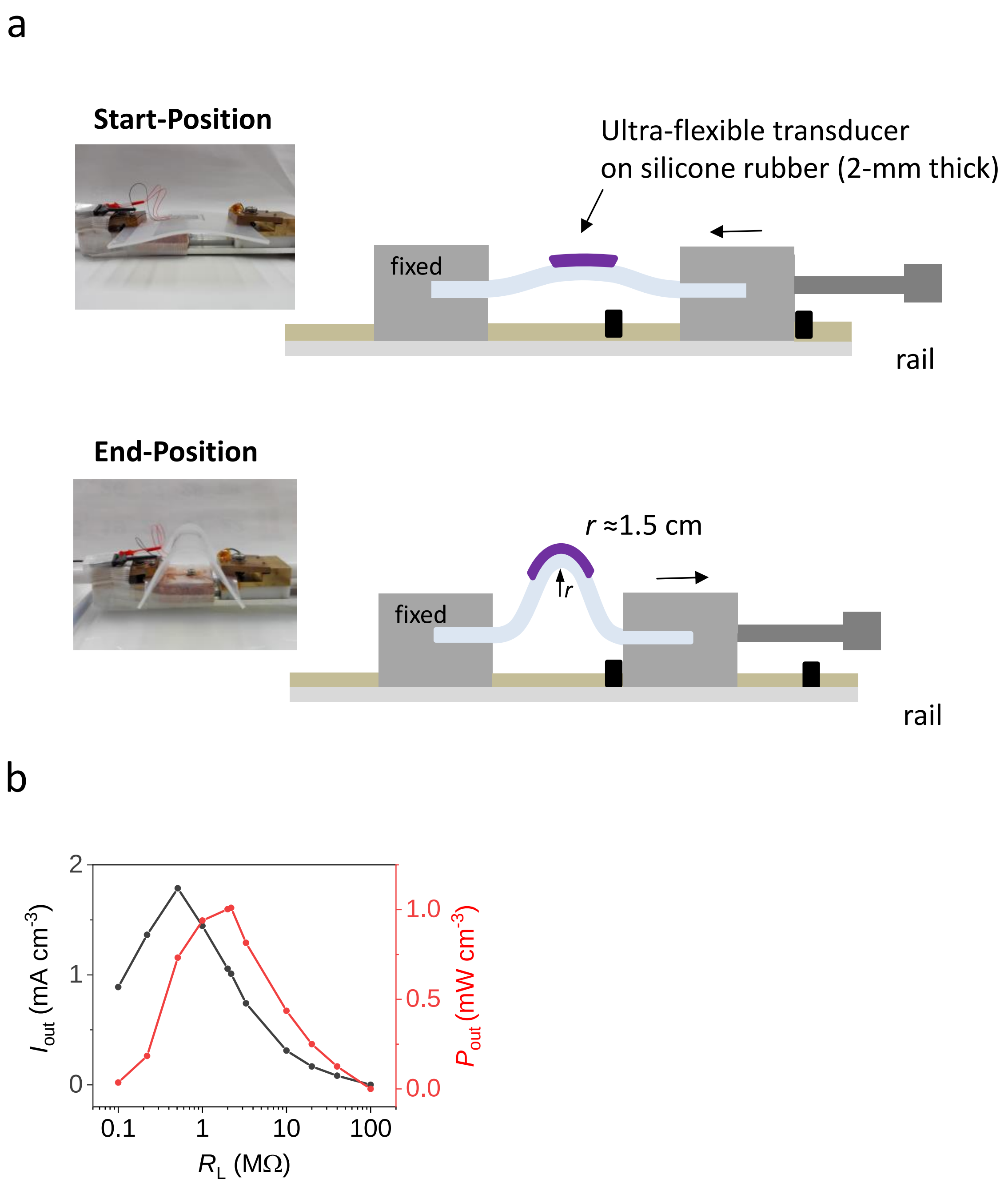

Supplementary Fig. 16 | Energy harvesting: UFPT bending tests on rail (mode B). a Schematic image and photographs of the bending test setup on a rail. The transducer is attached on the 2-mm thick silicone rubber film fixed between two clamps on a rail, whereby one side can be moved (typically by hand) between two pre-defined positions (mode B). **b** The current density I_{out} as well as the corresponding output power density P_{out} are plotted as a function of load resistance R_L for such an excitation scheme.

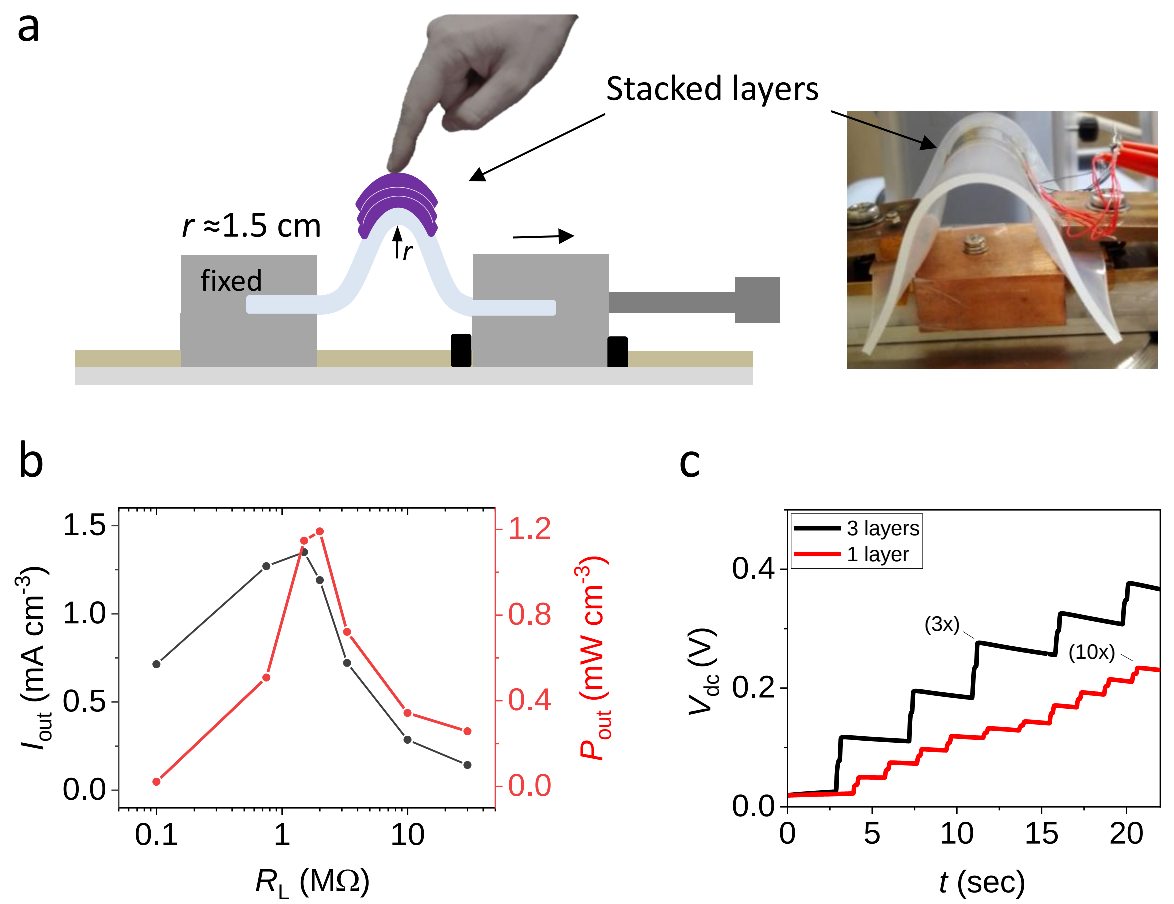

Supplementary Fig. 17 | Energy harvesting: Continuous pressing with a fingertip on the bent transducer (mode C). a Schematic image and photograph of the energy harvesting test setup showing multiple layers of transducers stacked on a curved silicone rubber carrier. Energy is generated by continuous touching on the PENGs (stacked PENGs are connected in parallel) (mode C). **b** The current density I_{out} as well as the corresponding output power density P_{out} are plotted as a function of load resistance R_L for continuous pressing with a fingertip on one bent transducer. **c** Loading voltages of a 1 µF capacitor corresponding to the energy generated with either a one- or three- stacked transducers during touching. For the triple-stack PENG three times (3x) slow touching delivers the same voltage on the capacitor as 10 times (10x) fast touching of a single layer PENG.

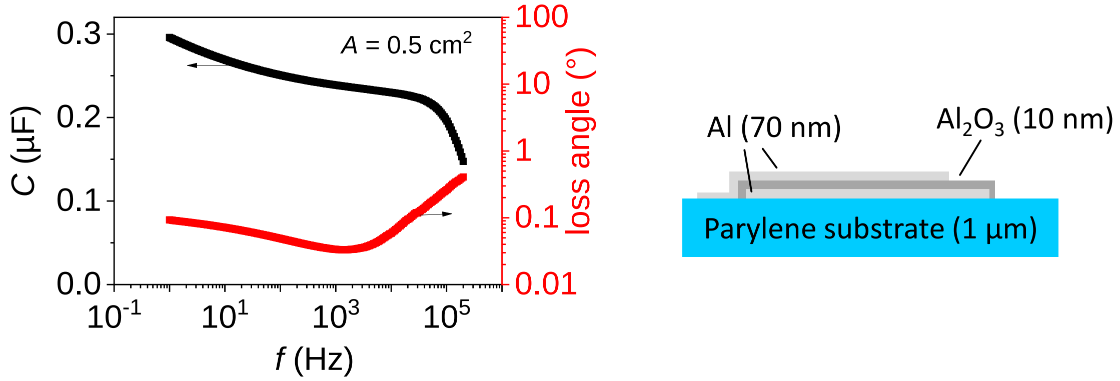

Supplementary Fig. 18 | Thin-film capacitor. Schematic image and LCR measurements of an Al (60–80 nm)/Alumina (10 nm, anodized)/Al (60–80 nm) thin-film capacitor.

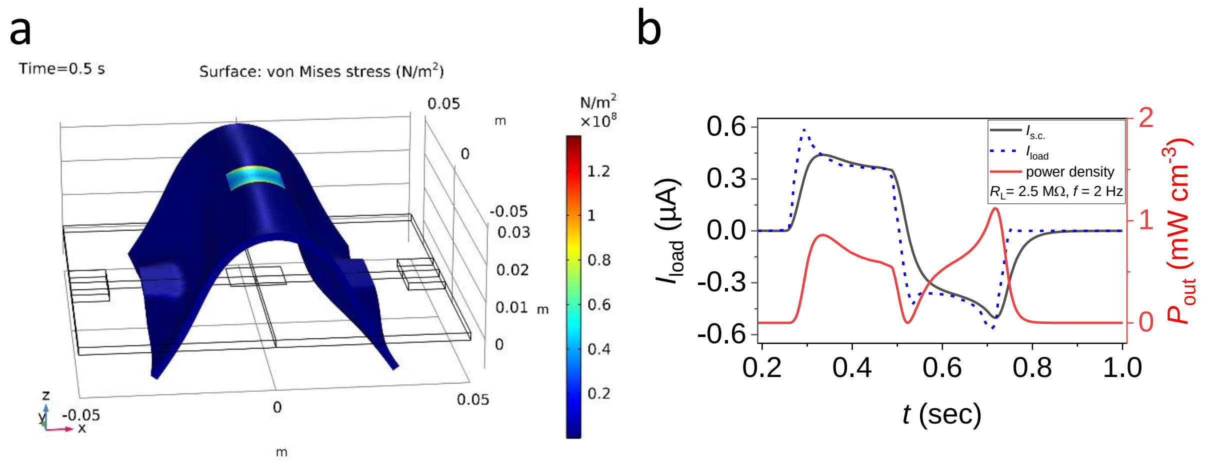

Supplementary Fig. 19 | FEM simulation of the bending actuation (mode B). a 3D representation of the model at full bending with stress levels in colour. **b** Time evolution of short circuit current ($I_{s.c.}$), load current (I_{load}) and calculated output power density for R_L = 2.5 MΩ.

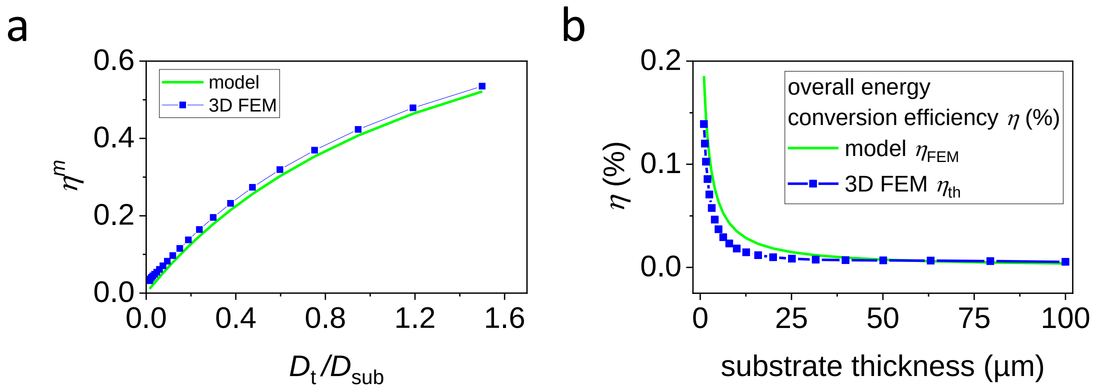

Supplementary Fig. 20 | Energy conversion efficiency of the UFPT (Mode B). Mechanical energy efficiency **a** and overall energy conversion efficiency **b** derived from the 3D FEM simulation and comparison with the analytical model for varying substrate thicknesses D_{sub}.

Supplementary Tables

Supplementary Table 1 | Transducer charge response under transversal load for the three excitation scenarios.

Carrier[a)]	Number of transducer layers	Transversal load			
		S_F (nC N^{-1}) [b)]	ΔF (N)[c)]	S_p (pC kPa^{-1})[b)]	Δp (kPa) [c)]
Glass (thickness = 6 mm, Y ~ 70 GPa)	1	0.054 (0.008)	0.2-10	11 (1.6)	1–50
Silicone rubber (thickness = 6 mm, Y = 1.45 MPa)	1	≈ 1.3	0.1–1	≈ 260	0.5–5
	2	≈ 2.5	0.1–1	≈ 520	0.5–5
Pre-bent silicone rubber (thickness = 6 mm, r = 1.5 cm)	1<br>2<br>3	≈ 4.8<br>≈ 9.7<br>≈ 14.8	2.5	n/a	n/a

[a)] Y: Young modulus; and r: bend radius. [b)] Sensitivity $S_F = \Delta Q/\Delta F$ (nC N^{-1}), and $S_p = \Delta Q/\Delta p$ (pC kPa^{-1}). [c)] Applied force ΔF and pressure Δp change range during transversal load tests. The sensitivity values on glass are mean values (calculated for six transducers), and standard derivations are indicated in brackets.

Supplementary Table 2 | Geometry and material parameters used for FEM analysis

Component	Part	Description	Symbol	Value
UFPT	Piezoelectric layer	Side length	L_t	15 mm
		Thickness	D_t	1.5 µm
		Young modulus	E_t	2.2 GPa
		Poisson ratio	v_t	0.28
		Remnant polarization	P_r	65 mC m^{-2}
	Parylene substrate	Side length	L_{sub}	15 mm
		Thickness	D_{sub}	{1, 2, 5} µm
		Young modulus	E_{sub}	2.8 GPa
		Poisson ratio	v_{sub}	0.40
		Side length	L_t	15 mm
Stamp		Side length	L_{sp}	10 mm
		Thickness	D_{sp}	0.4 mm
		Edge curvature	r_{sp}	0.2 mm
		Young modulus	E_{sp}	200 GPa
		Poisson ratio	v_{sp}	0.27
Rubber substrate		Side length	L_{sp}	20 mm
		Thickness	D_{sp}	6 mm
		Young modulus	E_{sp}	1.45 MPa
		Poisson ratio	v_{sp}	0.49

Supplementary Table 3 | Comparison of the performance parameter of vertical diodes and OTFT-based diodes, both with DNTT as the active semiconducting layer.

	Gate dielectric	OSC	J [a)] ($mA\ cm^{-2}$)	V_T [b)] (V)	Rectifying ratio[c)]	V_{break} [d)] (V)
Vertical diode with PFBT	-	DNTT	10^4 @ 5V	0.3–1.0	> 10^6	> −15
OTFT-based diode (W = 27mm)	AlO_x+ SAM	DNTT	105 @ 2V	< 0.1	$1.4 \cdot 10^7$ ($0.8 \cdot 10^7$)	> −5
OTFT-based diode (W = 7mm)	AlO_x+ SAM	DNTT	75 @ 2V	< 0.1	$3.6 \cdot 10^6$ ($3 \cdot 10^6$)	> −5
OTFT-based diode (W = 0.5mm)	AlO_x+ SAM	DNTT	65 @ 2V	< 0.1	$6.4 \cdot 10^5$ ($4.5 \cdot 10^5$)	> −5

[a)] Current density range of vertical Schottky and OTFT-based diodes at forward voltages of 5 V and 2 V, respectively. The channel length of the OTFTs is 12 µm, and the channel width varies between 0.5 mm and 27 mm; the area of the vertical diodes is 0.025 mm^2 and for the OTFT-based diodes, it is between 0.018 and 0.65 mm^2. [b)] V_T is the transition voltage; [c)] Rectifying ratio is defined as a ratio of the current in the 'on' (V = 5 V) and 'off' states (V = −5 V) for the vertical and ± 2 V for the OTFT-based diode. For the OTFT-based diodes with channel width of 0.5 mm, 7 mm and 27 mm the rectifying values are averaged over 15/10/6 devices, respectively, with the standard deviation values given in brackets; and [d)] V_{break} is the reverse breakdown voltage.

Supplementary References

1. Stadlober, B., Zirkl, M. & Irimia-Vladu, M. Route towards sustainable smart sensors: ferroelectric polyvinylidene fluoride-based materials and their integration in flexible electronics. *Chem. Soc. Rev.* **48**, 1787–1825 (2019).
2. Neumann, N., Köhler, R. & Hofmann, G. Infrared sensor based on the monolithic structure Si-P(VDF/TrFE). *Ferroelectrics* **171**, 225–238 (1995).
3. Furukawa, T. Ferroelectric properties of vinylidene fluoride copolymers. *Phase Transitions* **18**, 143–211 (1989).
4. Kuzumoto, Y. & Kitamura, M. Work function of gold surfaces modified using substituted benzenethiols: Reaction time dependence and thermal stability. *Appl. Phys. Express* **7**, 035701 (2014).
5. Liu, C., Xu, Y. & Noh, Y. Contact engineering in organic field-effect transistors. *Mater. Today* **18**, 79–96 (2015).
6. Kang, C. *et al.* 1 GHz Pentacene Diode Rectifiers Enabled by Controlled Film Deposition on SAM-Treated Au Anodes. *Adv. Electron. Mater.* **2**, 1500282 (2016).
7. Kang, C., Shin, H. & Lee, C. High-frequency organic rectifiers through interface engineering. *MRS Commun.* **7**, 755–769 (2017).
8. Moreno-Cruz, F. *et al.* treNch: Ultra-Low Power Wireless Communication Protocol for IoT and Energy Harvesting. *Sensors* **20**, 6156 (2020).
9. Singh, J., Kaur, R. & Singh, D. Energy harvesting in wireless sensor networks: A taxonomic survey. *Int. J. Energy Res.* **45**, 118–140 (2021).
10. Dagdeviren, C. *et al.* Recent progress in flexible and stretchable piezoelectric devices for mechanical energy harvesting , sensing and actuation. *Extrem. Mech. Lett.* **9**, 269–281 (2016).
11. IEEE Standard on Piezoelectricity, *ANSI/IEEE* 176-1987, (1988).
doi: 10.1109/IEEESTD.1988.79638
12. Furukawa, T. Piezoelectricity and pyroelectricity in polymers. *IEEE Trans. Electr. Insul.* **24**, 375–394 (1989).
13. Yamamoto, T. & Takimiya, K. Facile synthesis of highly pi-extended heteroarenes, dinaphtho 2,3-b : 2 ’,3 ’-f chalcogenopheno 3,2-b chalcogenophenes, and their application to field-effect transistors. *J. Am. Chem. Soc.* **129**, 2224 (2007).
14. Kumar, B., Kaushik, B. K. & Negi, Y. S. Perspectives and challenges for organic thin film transistors: materials, devices, processes and applications. *J. Mater. Sci. Mater. Electron.* **25**, 1–30 (2014).
