# Supplementary material for: Imperceptible energy harvesting device and biomedical sensor based on ultraflexible ferroelectric transducers and organic diodes
Source: Nat Commun. 2021 Apr 23;12:2399. doi: 10.1038/s41467-021-22663-6 (PMC8065095; doi:10.1038/s41467-021-22663-6)
Supplement: Supplementary file 2 — Description of Additional Supplementary Files [file 41467_2021_22663_MOESM2_ESM.docx]

**Description of Additional Supplementary Files**

File Name: **Supplementary Movie 1:**

Description: Demo movie of pulse wave measurement
